# Encapsulation of *Bifidobacterium bifidum* by internal gelation method to access the viability in cheddar cheese and under simulated gastrointestinal conditions

**DOI:** 10.1002/fsn3.1562

**Published:** 2020-04-14

**Authors:** Muhammad Afzaal, Farhan Saeed, Huda Ateeq, Aftab Ahmed, Awais Ahmad, Tabussam Tufail, Zoria Ismail, Faqir Muhammad Anjum

**Affiliations:** ^1^ Institute of Home & Food Sciences Government College University Faisalabad Faisalabad Pakistan; ^2^ The University of the Gambia Serrekunda Gambia; ^3^ University Institute of Diet & Nutrition Sciences Faculty of Allied Health Sciences, The University of Lahore Lahore Pakistan

**Keywords:** cheddar cheese, encapsulation, probiotic, stimulated gastrointestinal conditions

## Abstract

The current study was conducted to elucidate the impact of encapsulation on the stability and viability of probiotic bacteria (*Bifidobacterium bifidum*) in cheddar cheese and in vitro gastrointestinal conditions. Purposely, probiotics were encapsulated in two hydrogel materials (kepa carrageenan and sodium alginate) by using an internal gelation method. Cheddar cheese was supplemented with unencapsulated/free and encapsulated probiotics. The product was subjected to physicochemical (pH, titrable acidity, moisture, and protein) and microbiological analysis for a period of 35 days of storage. Furthermore, the probiotics (free and encapsulated) were subjected to simulated gastrointestinal conditions. The initial probiotic count in cheese containing encapsulated probiotic was 9.13 log CFU/g and 9.15 log CFU/g which decreased to 8.10 log CFU/g and 7.67 log CFU/g while cheese containing unencapsulated probiotic initially 9.18 log CFU/g decreased to 6.58 log CFU/g over a period of 35 days of storage. The incorporation of unencapsulated and encapsulated probiotic affected the physicochemical, microbiological, and sensory attributes of the cheese. The encapsulated probiotic bacteria exhibited better survival as compared to unencapsulated probiotic. A 2.60 CFU/g log reduction in unencapsulated cells while just 1.03 CFU/g and 1.48 CFU/g log reduction in case of sodium alginate and K‐carrageenan, respectively, was recorded. In short, encapsulation showed protection and stability to probiotic in hostile conditions.

## Graphical abstract

Microencapsulation technologies have wide application for the development of various functional foods, such as yoghurt, cheese, cultured cream, and frozen dairy desserts, and for biomass production encapsulation can be used for the target delivery of any sensitive ingredients. Encapsulation provides protection against pH changes, mechanical stress, temperature, enzymatic activity, time, and osmotic force.

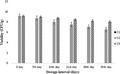

**Abbreviations**
KC: Kappa carrageenan
SA: Sodium Alginate
UE: Unencapsulated

## 1 INTRODUCTION

There is an emerging interest in the formulation and utilization of probiotics owing to their additional health benefits, and this motion has led probiotics to multimillion industry. On account of consumer awareness and adhered health claims, the demand for probiotic foods is growing momentously for day by day (Akter, Parvez, & Patwary, 2016). Probiotics are known to be microorganisms that, if present in appropriate amount in food, are able to withstand hostile conditions in gastrointestinal tract and adhere cells that promote positive effect (Hill et al., 2014). Moreover, they must be stable during processing and storage conditions.

Several researches have proved the sustainability of the probiotics in any food product not only depends on the storage of food but on food matrix components like fat, protein, and/moisture contents too. The minimum count of microorganisms should be 10^6^–10^7^ CFU/ml or CFU/g viable cells at the time of intake (Castro, Tornadijo, Fresno, & Sandoval, 2015).

The potential probiotics that are generally used in functional dairy products belong to the genera Lactobacillus and Bifidobacterium (Ranadheera, Naumovski, & Ajlouni, 2018). Among these, *Lb. reuteri* and *B. animalis* subsp. lactis are the most suggested to be used in food.

There is an emerging interest in the development of dairy food products that have lactic acid bacteria (LAB) and bifidobacteria. These bacteria prevent the growth of other microorganisms by producing different metabolites including organic acids, alcohol compounds, bacteriocins, and diacetyl (Buriti, Cardarelli, & Saad, 2007).

Cheese is one of the most effective food matrices for maintaining viable probiotic bacteria and integrating into human nutrition (Caggia, De Angelis, Pitino, Pino, & Randazzo, 2015; Thomas, 2016). Combination of *Bifidobacterium* and *Lactobacillus* (single or mixed culture) in cottage cheese (Abadía‐García et al., 2013), Minas fresh cheese (Verruck et al., 2015), pasta filata soft cheese (Cuffia et al., 2017), and mascarpone cheese (de Almeida et al., 2018) are most preferred for or their better viability in carrier foods (Phillips, Kailasapathy, & Tran, 2006). The aptness of cheese for providing a number of probiotics, that is, Bifidobacteria, *L.paracasei*, and *L.acidophilus*, has been stated by Alves et al. (2013) and Santini et al. (2012).

The inoculation of probiotics in cheese‐making process faces many challenges, and the most important is the maintenance and survival of beneficial bacteria during handling and storage and maintenance of sensory characteristics (Murtaza, Huma, Shabbir, Murtaza, & Anees‐ur‐Rehman, 2017; Tomar, 2019). For the maintenance of probiotic living cell, a physical barrier against adverse external conditions is receiving considerable interest. Research inclinations are moving toward the use encapsulation to improve and sustain the viability of probiotics throughout the storage conditions. Entrapping microorganisms in a casing (Encapsulation) is an efficient method to ease the damage of probiotic storage (Krasaekoopt, Bhandari, & Deeth, 2006) stabilizes cells, potentially enhancing their viability and stability in the production, storage, and handling of probiotic cultures (Tripathi & Giri, 2014).

Therefore, this study was aimed to probe the effect of viability of encapsulated probiotics in cheddar cheese over a period of 35 days of storage conditions and its effect on gastrointestinal conditions, sensory profile, and textural characteristics was evaluated.

## 2 MATERIALS AND METHODS

Good quality raw milk was purchased from an indigenous farm for the preparation of the cheddar cheese. Probiotic culture (*Bifidobacterium bifidum)* and enzyme (rennet) were obtained from NIFSAT, University of Agriculture Faisalabad. Kepa‐carrageenen and sodium alginate were purchased from scientific store. The research was carried out at Food Safety and Biotechnology laboratory, Government College University, Faisalabad.

### 2.1 Culture activation

Pure freeze‐dried culture of *Bifidobacterium bifidum* (ATTC‐29521) was obtained from NIFSAT, University of Agriculture Faisalabad, Pakistan. Probiotic cells were activated by inoculating it in MRS (Man Rogosa Sharpe) broth at 37°C for 24 hr. Afterward, the cells were centrifuged in a centrifuge machine (75005276 EA, Thermo Fisher Scientific Inc.). The obtained probiotic cells were encapsulated for further studies.

### 2.2 Encapsulation of *B. bifidum*

*Bifidobacterium bifidum* was encapsulated with k‐carrageenan and sodium alginate microgels by following the method as described by Afzaal et al. (2018) and Mokhtari *et al.* (2019) with little modifications. Shortly, 100 ml of 3% (w/v) k‐carrageenan and sodium alginate solutions was prepared and autoclaved. The prepared solutions were mixed with concentrated probiotic culture (10^10^ CFU/ml). The solution of both wall materials was then distributed by using 5 ml syringe into a beaker containing oil and Tween 80 solution as an emulsifier. The obtained mixture was stirred at 150 rpm with a help of magnetic stirrer. Solution of calcium chloride was used to brake the emulsion. The obtained microbeads were washed with double distilled water and stored at refrigerated temperature.

### 2.3 Encapsulation yield

The encapsulation yield was calculated by using the method of Iqbal, Zahoor, Huma, Jamil, and Ünlü (2018). For this purpose, 20 microbeads were selected randomly from both type of encapsulated formulations.

The selected beads were disintegrated using a stomacher bag containing a phosphate buffer solution and a solution of sodium citrate having a molarity of 0.1 M at pH of 6.3 by using a stomacher bag. The number of viable cells coated in SA and KC was determined by using pour plate technique. The yield was calculated by using the following formula:

EY = Number of cells released (sodium alginate bead and k‐ carrageen bead) × 100.

Number of cells added (sodium alginate and k‐carrageenan solution).

### 2.4 Cheddar cheese preparation

Cheddar cheese was made by following the method of Czárán, Rattray, Cleide, and Christensen (2018) with a little modification. Raw milk was standardized at 3.5% fat. Milk was pasteurized at 65°C for 15 min, and afterward it was acidified using rennet and starter culture was added in it. Afterward, it was incubated at 37°C for 4 hr. The cheese was divided into three different categories, that is, control, free probiotic, and encapsulated (k‐carrageenan and sodium alginate) as shown in Table 1.

**Table 1:** Treatment plan for cheddar cheese preparation

Trails	Type
C1	No probiotic (control)
C2	Cheese containing unencapsulated probiotic bacteria
C3	Cheese containing microbeads (encapsulated with sodium alginate)
C4	Cheese containing microbeads (K‐carrageenan encapsulation)

The samples were wrapped in small packages and stored for 35 days at 4°C. The experimentation was performed in triplicates. The product was subjected to physicochemical and microbiological analysis.

### 2.5 Physicochemical analysis of cheddar cheese

Cheddar cheese from all cheese treatments was analyzed in triplicates for pH, moisture, protein, and fat content by following AOAC method (2006). pH was determined by a digital pH meter. Moisture contents were determined by drying oven method. Titrable acidity and protein were estimated by titration method and Kjeldahl method, respectively.

### 2.6 Probiotic viability in cheese

Viability of *B. bifidum* in cheddar cheese was examined at 0 day of storage and continued for 35 days with an interval of 7 days. The sample cheese was mixed with peptone water (0.1%), and it was diluted up to 108 with the similar diluent in a stomacher bag. The viability of free and encapsulated probiotics was evaluated by method as described by Sohail, Turner, Prabawati, Coombes, and Bhandari (2012). Bacterial colonies were observed and calculated after incubation period as described by Mokarram, Mortazavi, Najafi, and Shahidi (2009).

### 2.7 Sensory analysis

Sensory evaluation of all cheese samples was carried out by the method of García‐Gómez, Romero‐Rodríguez, Vázquez‐Odériz, Muñoz‐Ferreiro, and Vázquez (2019). Each panelist received cheese sample in plate which was coded with an arbitrary alphabet, and samples of all the four types of cheese were simultaneously served in a random sequence.

All the panelists were asked to select the most preferred cheese sample to evaluate results. They were asked to take a sip of water in between the samples. A nine hedonic scale was used with 1 (dislike the most) to 9 (like the most). Results were noted on the sensory evaluation sheet for all the parameters which includes color, taste, appearance, texture, and general perception.

### 2.8 In vitro gastrointestinal assay

Viability of free and encapsulated cells in simulated and intestinal conditions was determined as described by Damodharan, Palaniyandi, Yang, and Suh (2017). Simulated gastric condition was prepared by making a low pH (~2). The pH was adjusted by the addition of 5M HCL. Free and encapsulated cells of S.A and K.C were added to the test tubes and incubated at 37°C. The viability of free and encapsulated cells was recorded at 0, 30, 60, 90, and 120 min. Similarly, viability and stability in simulated intestinal conditions were determined by adjusting pH to 7.5. Free and encapsulated cells were added to test tubes containing the solution, and results were recorded at defined intervals (0, 30, 60, 90, and 120 min).

### 2.9 Statistical analysis

All the data were directly subjected to ANOVA (analysis of variance) to observe the significant difference (*p* < .05) between the cheddar cheese treatments. The results were stated as the mean values from the three replicates.

## 3 RESULTS AND DISCUSSION

### 3.1 Encapsulation yield

Wall materials have direct effect on the encapsulation yield. The encapsulation yield also affects the stability in simulated digestive conditions (Shi et al., 2013). A difference in encapsulation yield was observed between both types of wall materials. High encapsulating yield was observed for S.A as compared to K.C. A comparison of both encapsulating material is shown in Table 2.

**Table 2:** Encapsulation efficiency

Type of coating matrix	Numbers before encapsulation	Numbers after encapsulation	Efficiency (%)
Sodium Alginate	8.73 ± 0.09	8.42 ± 0.03	96
Kepa Carrageenan	8.39 ± 0.04	7.87 ± 0.06	93

### 3.2 Physicochemical analysis of cheddar cheese

#### 3.2.1 Effect probiotics supplementation (free and encapsulated) on pH of cheese

**Figure 1:**
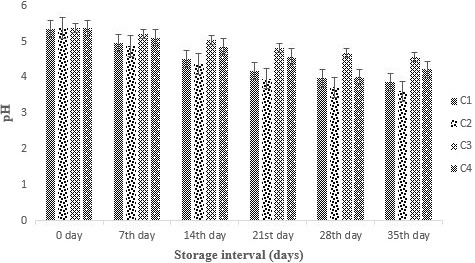
Effect of free and encapsulated (with sodium alginate and carrageenan) on pH of cheddar cheese during storage intervals (0, 7, 14, 21 28, and 35 days) compared with control. Each bar represents mean value for pH of treatments. C1 (Control without addition of probiotics), C2 (Free/unencapsulated cells), C3 (Probiotics encapsulated with sodium alginate), and C4 (Probiotics encapsulated with carrageenan)

The pH of probiotic food has a direct relationship with the stability of the probiotic bacteria. A decreasing trend in all type of cheese samples was observed shown in Figure 1. The findings of the present study indicated that cheese containing encapsulated *B. bifidum* showed a slow reduction in pH as compared to the cheese containing free/unencapsulated probiotics. The results are in accordance with Batista et al. who found that pH decrease with storage (Batista et al., 2017).

#### 3.2.2 Effect of probiotic supplementation (free and encapsulated) on titrable acidity of cheese

**Figure 2:**
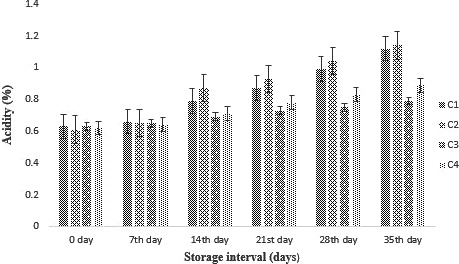
Effect of free and encapsulated probiotics on titrable acidity of cheddar cheese during storage intervals (0, 7, 14, 21 28, and 35 days) compared with control. Each bar represents mean value for titrable acidity of treatments. C1 (Control without addition of probiotics), C2 (Free/unencapsulated cells), C3 (Probiotics encapsulated with sodium alginate), and C4 (Probiotics encapsulated with carrageenan)

The results regarding the titrable acidity of cheddar cheese are shown in Figure 2. An increasing trend was observed for acidity of cheddar cheese. The maximum titrable acidity was observed for cheese containing free probiotic (UE). Probiotic bacteria consume the lactose content of the milk and cheese and produce organic acids which results in lower pH and increases the acidity as discussed by Batista et al. (2017).

#### 3.2.3 Effect of probiotics supplementation (free and encapsulated) on moisture content

**Figure 3:**
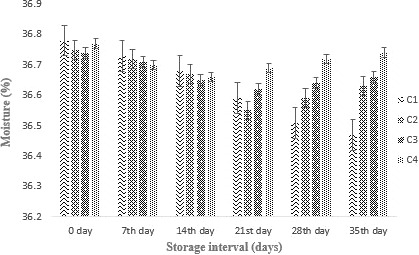
Effect of free and encapsulated on moisture of cheddar cheese during storage intervals (0, 7, 14, 21 28, and 35 days) compared with control. Each bar represents mean value for moisture of treatments. C1 (Control without addition of probiotics), C2 (Free/unencapsulated cells), C3 (Probiotics encapsulated with sodium alginate), and C4 (Probiotics encapsulated with carrageenan)

All cheddar cheese samples showed a minor decrease in moisture content throughout storage for over 35 days as shown in Figure 3. After storing it for 35 days, no significant change in moisture content was recorded. However, the lowest moisture content was found in treatment C (control). The results were in line with Ningtyas, Bhandari, Bansal, and Prakash (2019).

#### 3.2.4 Effect of probiotic supplementation (free and encapsulated) on protein content

**Figure 4:**
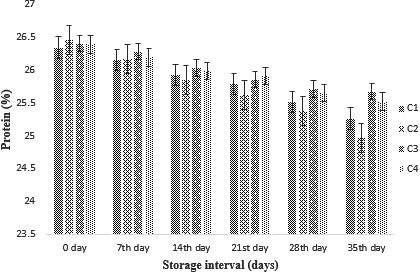
Effect of free and encapsulated on protein content of cheddar cheese during storage intervals (0, 7, 14, 21 28, and 35 days) compared with control. Each bar represents mean value for protein content of treatments. C1 (Control without addition of probiotics), C2 (Free/unencapsulated cells), C3 (Probiotics encapsulated with sodium alginate), and C4 (Probiotics encapsulated with carrageenan)

The results from the protein content of cheddar cheese were found to be significant. A decreasing trend was obtained as shown in Figure 4. The decreasing trend in protei(n may be due to metabolic activities of probiotics. A rapid decrease was observed in UE (treatment with free probiotic/ unencapsulated), but SA (encapsulated with sodium alginate) showed the lowest decrease in the protein content of cheese.

#### 3.2.5 Effect of encapsulation on the viability and stability of probiotic in cheese

**Figure 5:**
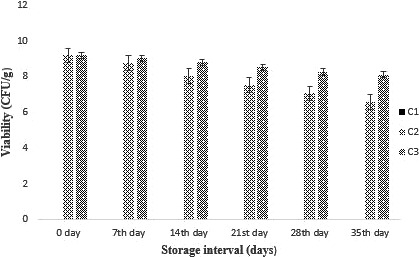
Viability of free and encapsulated probiotics in cheddar cheese during storage intervals (0, 7, 14, 21 28, and 35 days). Each bar represents mean value for the viable count for treatments.C2 (Free/unencapsulated cells), C3 (Probiotics encapsulated with sodium alginate), and C4 (Probiotics encapsulated with carrageenan)

Overall, a decreasing tendency was observed for the viability of probiotics (unencapsulated and encapsulated). Probiotic viability and stability as a carrier food are of great importance. A slow decline in log reduction was observed in cheese sample containing encapsulated probiotics as compared to nonencapsulated cells as shown in Figure 5. A log reduction of 2.60 log CFU/g was noted in case of nonencapsulated probiotic cells while just 1.03 log CFU/g and 1.48 CFU/g reduction was observed in cheese sample encapsulated with probiotic bacteria. The results explained that encapsulation ensures that stability and viability of probiotic bacteria.

The initial *B. bifidum* population in cheddar cheese samples was about 9.18 log CFU/g that dropped uninterruptedly over 35 days of storage. Cheese treatments SA (encapsulated with sodium alginate) and KC (encapsulated with K‐carrageenan) showed a comparatively more number of probiotic viable cells than nonencapsulated cheese treatment UE (unencapsulated probiotic). However, a small reduction in viable count was observed in cheese treatment C2 (treatment with free probiotic) which reduces from 9.13 log CFU/g to 8.1 log CFU/g. In a research held by Gbassi, Vandamme, Ennahar, and Marchioni, (2009) enhanced survivability of *L. plantarum* was detected in simulated digestive conditions as it was coated in alginate. Therefore, to sustain the survival of probiotics throughout storage period, encapsulation procedure is an efficient technique. Furthermore, after storage of 35 days, all cheese treatments exhibited an endurable population of *B. bifidum* (>10^6^ CFU/g) at a value above than 10^6^ CFU/g that is proposed to provide probiotic benefits as suggested by Castro et al. (2015). From the results, it can be suggested that cheddar cheese is a potential carrier of probiotic bacteria.

#### 3.2.6 Effect of prebiotic supplementation on sensory attributes of cheese

**Figure 6:**
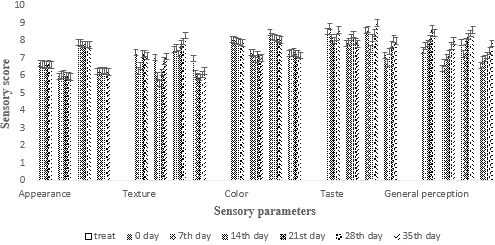
Effect of free (unencapsulated) and encapsulated (with sodium alginate and carrageenan) on sensory characteristics of cheddar cheese during storage intervals (0, 7, 14, 21 28, and 35 days) compared with control. Each bar represents mean value for sensory scores for sensory parameters of cheese. C1 (Control), C2 (Free/encapsulated cells), C3 (Probiotics encapsulated with sodium alginate), and C4 (Probiotics encapsulated with carrageenan)

Sensory quality of food products is thought out as the leading parameter as it demonstrates the consumer's preferences (Albenzio et al., 2013). The food manfacturing industry need to know what people like and dislike about their product through the sensory evaluation (Popper, Rosenstock, Schraidt, & Kroll, 2004). In this study, sensory evaluation of all the cheddar cheese treatments was done according to 9‐hedonic scale and the mean results of all the parameters were shown in Figure 6. All the results from sensory analysis were found to be significant except color and appearance which showed nonsignificant results.

When texture of all the cheese treatments was observed, the results of SA (encapsulated with sodium alginate) showed highest scores as shown in Figure 6, whereas C (control sample) showed less increase in this parameters. However, previous studies did not noticeably change the sensory profile of cheese (Albenzio et al., 2013).

Taste is considered as one of most important constituent in all the food products. Mean results of taste parameter were shown in Figure 6. The control sample C (control treatment) and treatment SA (encapsulated with sodium alginate) showed maximum score. Results of KC (encapsulated with K‐carrageenan) were found quite close to SA (encapsulated with sodium alginate).

No significant increase in results was obtained for color and appearance as shown in Figure 6. However, appearance of treatment SA (encapsulated with sodium alginate) containing encapsulated probiotic highest( results. Similarly, the color of all cheese treatments was found to be nonsignificant. The results obtained for control treatment C (control treatment) and that of SA (encapsulated with sodium alginate) were quite close. However, SA (encapsulated with sodium alginate) had high points in contrast to all the treatments. Similar results were found by Oliveira et al. (2012), in which probiotic lactic acid bacteria were added in goat cheese and no significant increase in results was found for color and appearance. Similarly, Yerlikaya and Ozer (2001) found no effect on the appearance of cheese with *S. thermophilus*.

The results regarding general perception of all cheese treatments were found to be significant. C (control treatment) shows the highest points. More research studies might suggest the same results.

#### 3.2.7 Viability and stability of encapsulated probiotics in simulated gastric conditions

**Figure 7:**
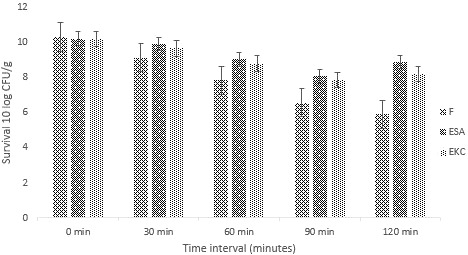
Survival of free and encapsulated probiotics under stimulated gastric condition during storage intervals of (0, 30, 60, 90, and 120 min). Each bar represents mean value for simulated intestinal condition. *F* (Free probiotics), ESA (Probiotics encapsulated with sodium Alginate), and EKC (Probiotics encapsulated with k‐carrageenan)

The stability and viability of probiotic bacteria are important in GIT. Feasibility of probiotic cells is vital in stomach and intestinal conditions so that the desired benefits of probiotics can be achieved. The probiotic cells (nonencapsulated and encapsulated) were subjected to gastric juice. A rapid log reduction was observed for nonencapsulated bacteria in contrast to encapsulated probiotic cells. Encapsulation of SA (encapsulation with sodium alginate coating) results better for the survival of probiotics as compared to KC (encapsulation with K‐carrageenan coating) encapsulation as shown in Figure 7. The results confirmed that encapsulation has a shielding effect toward probiotics in simulated gastric conditions.

#### 3.2.8 Stability and viability of encapsulated probiotics in intestinal conditions

**Figure 8:**
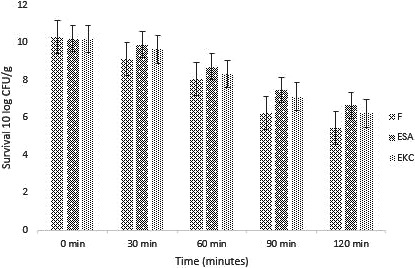
Survival of free and encapsulated probiotics under simulated intestinal conditions during storage intervals of (0, 30, 60, 90, and 120 min). Each bar represents mean value for simulated intestinal condition. *F* (Free probiotics), ESA (Probiotics encapsulated with sodium Alginate), and EKC ( (Probiotics encapsulated with k‐carrageenan)

Wall materials which are dissimilar showed a shielding result on probiotics after they were exposed to the intestinal conditions. Current study showed probiotics in free (unencapsulated) and encapsulated forms were added in artificial simulated intestinal solution for a defined time period. A sudden drop in probiotic which was without encapsulation was observed in contrast to the encapsulated cells at 7.5 pH, as shown in Figure 8. The encapsulated probiotic with both sodium alginate and K‐carrageenan showed a significant effect (*p* < .05) on the survival of the cells. The sodium alginate encapsulated beads and K‐carrageenan encapsulated bead cells showed a gentle log reduction when compared to the cells in free form. The current study is in line with Iqbal et al. (2018). They stated that encapsulation of cells with alginate enhanced the discharge and viability of probiotic bacteria in GIT conditions. From the results, it was revealed that encapsulation is an essential technique for durability of subtle components.

## 4 CONCLUSION

The current research was conducted to elucidate the effect of probiotics in unencapsulated (free) and encapsulated form. Encapsulation ensured a better viability of probiotic bacteria in a carrier food and survive better in stimulated gastrointestinal conditions. As a coated material sodium alginate showed better results as compared to K‐carrageenan. It can be concluded that internal gelation encapsulation technique can be used to sustain the suggested level (<10^6^–10^8^) of probiotics in carrier food.

## CONFLICT OF INTEREST

Authors declare that they have no conflict of interest.

## ETHICAL APPROVAL

This article does not contain any studies with human participants or animals performed by any of the authors.

## INFORMED CONSENT

For this type of study, formal consent is not required.

5
